# In this issue: Volume 115, Issue 2, February 2024

**DOI:** 10.1111/cas.16084

**Published:** 2024-02-11

## Oral bacterium *Streptococcus mutans* promotes tumor metastasis through thrombosis formation

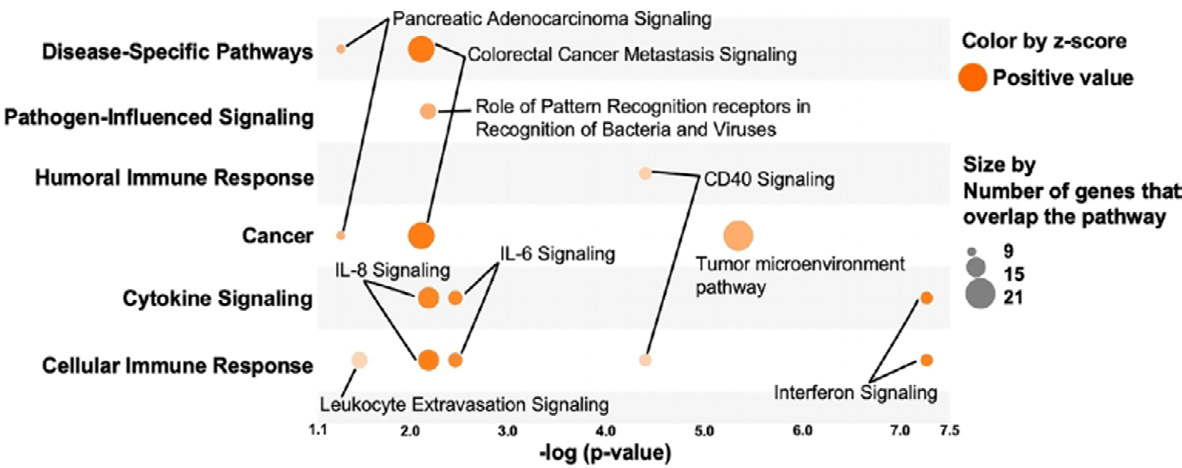

Bacteria that colonize the human mouth are known to enter the bloodstream due to oral diseases, such as periodontitis. Clinical evidence suggests that oral bacteria in the blood increase the incidence of cardiovascular complications and promote the migration or ‘metastasis’ of cancer cells. The oral bacterium *Streptococcus mutans* is known to invade the inner cell layer of blood vessels, called the endothelium, leading to inflammation. Inflammation in the blood vessels further leads to blocked blood flow or ‘thrombosis,’ the main known mechanism through which oral bacteria induce cardiovascular complications. However, whether *S. mutans*‐induced inflammation and resultant thrombosis promote the progression of cancer remains unclear.

Through a recent study conducted to address this knowledge gap, Yu et al. confirm that *S. mutans* promotes metastasis of cancer cells via thrombosis in a mouse model of breast cancer. In their study, the researchers found that *S. mutans* caused inflammation in the endothelial cells, activated platelets, and caused them to aggregate in these cells, leading to thrombosis in mice that received intravenous injections of the bacterium. Furthermore, *S. mutans* increased the chance of tumor cells in the bloodstream sticking to inflamed endothelial cells, resulting in a greater risk of new tumors in the lungs. Administering aspirin—an antiplatelet drug that prevents thrombosis—reduced breast cancer metastasis in the lungs of the tested mice.

The findings of this study suggest that the oral bacterium in the bloodstream may promote the metastasis of cancer to other parts of the body. Specifically, this study offers compelling evidence that *S. mutans* may increase the chance of breast cancer spreading to the lungs. Clinicians may consider incorporating aspirin into the treatment of cancers to prevent the risk of thrombosis and reduce the cancer‐promoting effects of blood‐borne oral bacteria. These findings also suggest that oral health influences the risk of cardiovascular complications and cancer metastasis. Therefore, oral care by dentists may help prevent deaths from cardiovascular diseases and malignant cancer.

https://doi.org/10.1111/cas.16010

## Inhibiting cholesterol de novo synthesis promotes hepatocellular carcinoma progression by upregulating prostaglandin E synthase 2‐mediated arachidonic acid metabolism under high fatty acid conditions

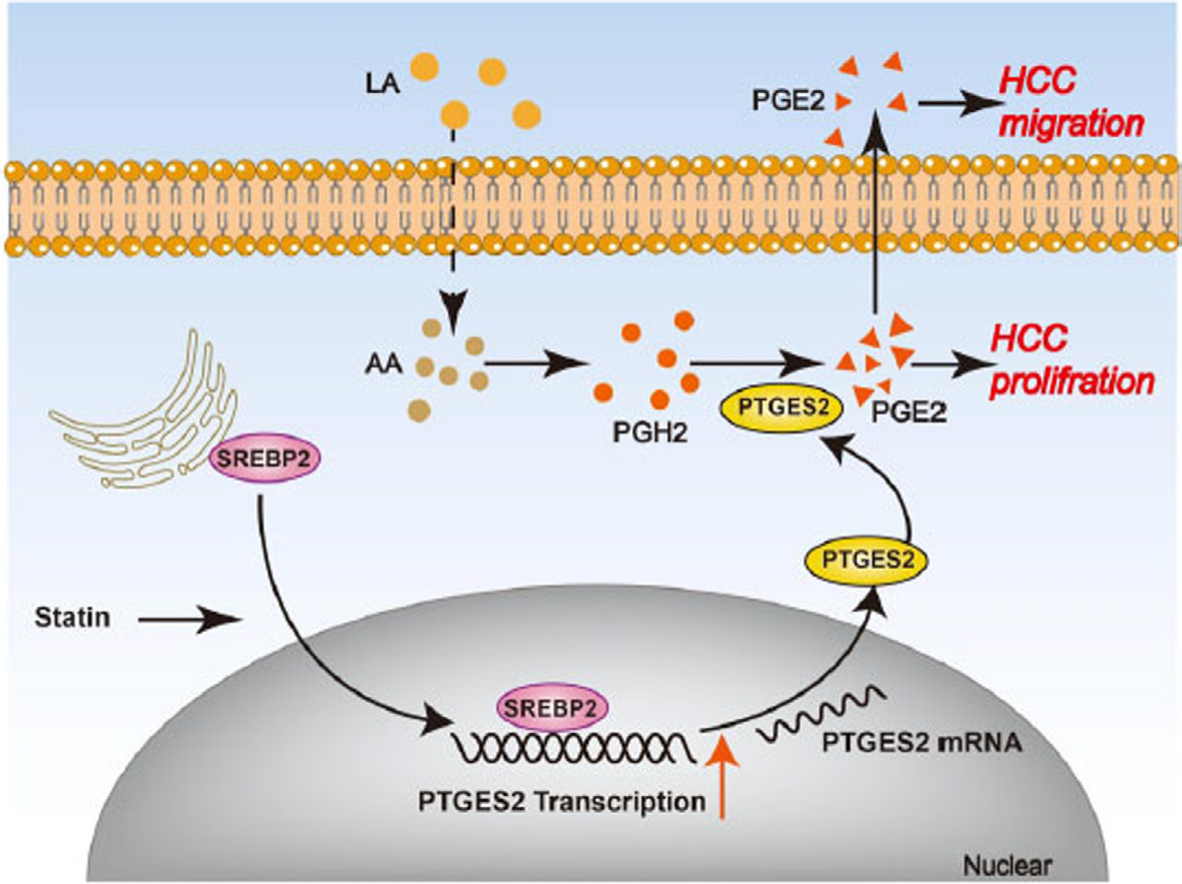

Hepatocellular carcinoma (HCC) represents a prevalent and life‐threatening type of liver cancer. HCC can significantly elevate the synthesis of cholesterol by the liver, negatively impacting the patient’s overall health. To prevent the overproduction of cholesterol by the affected liver, treatment of HCC sometimes involves the use of drugs called statins, which help regulate cholesterol synthesis. However, the impact of these drugs on the advancement of HCC remains unclear, especially under conditions where the patient’s liver shows elevated fatty acid levels.

This study explored the role of atorvastatin, a commonly used statin, in proliferation and migration of HCC tumor cells. The study was conducted on mice fed with a diet rich in fatty acids. It was found that atorvastatin promotes cancer progression by interfering with the arachidonic acid (AA) metabolism pathway. Atorvastatin increases the expression of prostaglandin E synthase 2 (PTGES2), a key enzyme involved in AA metabolism. Additionally, the study found that atorvastatin’s did not exert any cancer‐promoting effects in mice in the absence of PTGES2 or linoleic acid, which is the precursor molecule to AA.

Finally, atorvastatin demonstrates an increase in the expression of PTGES2, which elevates the levels of AA in the liver and ultimately promotes cancer progression. Our study provides preliminary evidence for the cancer‐promoting effect of atorvastatin in HCC under high fatty acid conditions. Therefore, the use of atorvastatin in HCC treatment may negatively affect the well‐being of patients with elevated fatty acid levels in the liver. Therefore, targeting the AA metabolism pathway or its components, such as linoleic acid or the PTGES2 enzyme, may help reduce the adverse side effects of statin treatment in HCC patients with fatty liver. However, a combinatorial approach involving both the inhibition of cholesterol synthesis using statins and the knockdown of AA metabolism may offer the best results during HCC treatment.

https://doi.org/10.1111/cas.16035

## Cross‐talk between gastric cancer cells and hepatic stellate cells promotes invadopodia formation during liver metastasis

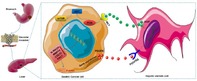

Gastric cancer (GC) is one of the most common causes of deaths worldwide. It is usually diagnosed late, when the cancer has progressed to an advanced stage, or the patient has distant metastases. Distant metastasis is when the primary tumor, i.e., tumor from the original site, spreads to other parts of the body. It mostly affects the liver in patients with GC. In patients with GC with liver metastasis (GCLM), the survival rate is poor, regardless of interventions like surgery and chemotherapy. Complicating matters, the development of GCLM remains unclear.

It is known that during metastasis, tumor cells lose their surrounding extracellular matrix (ECM)—a complex network of molecules that provides structural support to the cells. Moreover, tumor cells form invadopodia, which are finger‐like membrane protrusions, to degrade and escape the ECM. Could invadopodia be involved in GCLM development?

To find out, Ren et al. conducted a study using tumor‐, liver metastatic‐, and normal paracancerous (located near cancer) tissues from 54 patients with GCLM. They intended to study the communication pathways between GC cells and hepatic stellate cells (HSCs), which, on activation, promote liver cancer by secreting cell‐signaling cytokines that influence tumor growth and metastasis.

The authors discovered that invadopodia‐specific proteins were significantly more abundant in metastatic liver tissues compared to the primary tumors of patients with GCLM. Additionally, the GC cells secreted platelet‐derived growth factor subunit B (PDGFB) that bound to platelet‐derived growth factor receptor beta (PDGFRβ) on HSCs and activated them. The activated HSCs secreted the cytokine hepatocyte growth factor (HGF), which enhanced the expression of the MET proto‐oncogene—a gene that can induce cancer when expressed abnormally—in the GC cells. Subsequently, PI3K/AKT signaling induced by MET facilitated the formation of significantly more invadopodia on GC cells in liver metastasis when compared to those in primary tumors, thereby aggravating GC metastasis.

These findings suggest that this crosstalk between cancer cells in the stomach and liver promotes invadopodia formation and exacerbates GC cell metastasis through the PI3K/AKT signaling pathway. This crosstalk between GC cells and HSCs can be used as a viable target for developing new therapies to treat patients with GCLM.

https://doi.org/10.1111/cas.16023

